# Why is* Babesia* not killed by artemisinin like* Plasmodium*?

**DOI:** 10.1186/s13071-023-05783-4

**Published:** 2023-06-08

**Authors:** Wenwen Si, Chuantao Fang, Chuang Liu, Meng Yin, Wenyue Xu, Yanna Li, Xiaoli Yan, Yujuan Shen, Jianping Cao, Jun Sun

**Affiliations:** 1 Institute for Infectious Diseases and Vaccine Development, School of Medicine, Tongji University, Shanghai, People’s Republic of China; 2 National Institute of Parasitic Diseases, Chinese Center for Disease Control and Prevention (Chinese Center for Tropical Diseases Research), Shanghai, People’s Republic of China; 3 Department of Pathogenic Biology, Army Medical University (Third Military Medical University), Chongqing, People’s Republic of China; 4 Present Address: Shanghai Tenth People’s Hospital, Tenth peoples hospital of Tongji university, Shanghai, People’s Republic of China

**Keywords:** *Babesia*, *Plasmodium*, Artemisinin, Pentose phosphate pathway, Single-cell sequencing, Haem synthesis

## Abstract

*Babesia* spp. are intraerythrocytic apicomplexans that digest and utilize red blood cells in a similar way to intraerythrocytic *Plasmodium* spp., but unlike the latter, are not sensitive to artemisinin. A comparison of *Babesia* and *Plasmodium* genomes revealed that *Babesia* genomes, which are smaller than those of *Plasmodium*, lack numerous genes, and especially haem synthesis-related genes, that are found in the latter. Single-cell sequencing analysis showed that the different treatment groups of *Babesia microti* with expressed pentose phosphate pathway-related, DNA replication-related, antioxidation-related, glycolysis-related, and glutathione-related genes were not as sensitive to artemether as *Plasmodium yoelii* 17XNL. In particular, pentose phosphate pathway-related, DNA replication-related, and glutathione-related genes, which were actively expressed in *P. yoelii* 17XNL, were not actively expressed in *B. microti*. Supplying iron in vivo can promote the reproduction of *B. microti*. These results suggest that *Babesia* spp. lack a similar mechanism to that of malaria parasites through which the haem or iron in hemoglobin is utilized, and that this likely leads to their insensitivity to artemisinin.

**Graphical Abstract:**

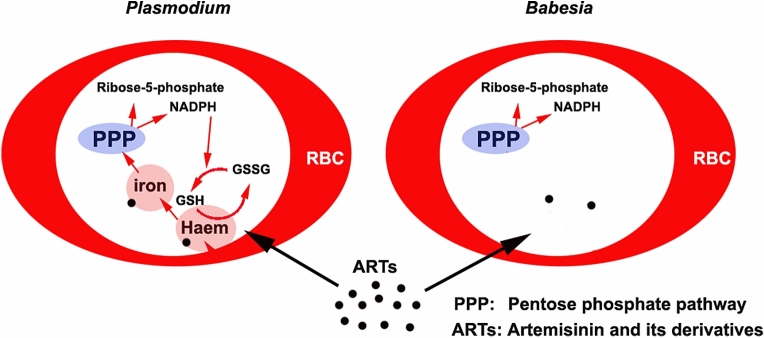

**Supplementary Information:**

The online version contains supplementary material available at 10.1186/s13071-023-05783-4.

## Background

The hemolytic disease babesiosis is caused by species of the protozoan parasitic genus *Babesia*. More than 100 species of *Babesia* have been identified, of which a few have been shown to be pathogenic to humans [1]. Over 100 species of *Plasmodium* have also been identified, of which five cause malaria in humans. Both *Babesia* and *Plasmodium* are intraerythrocytic protozoa and are associated with common clinical signs such as hemolytic anemia, fever, choloplania, hemoglobinuria, and an enlarged spleen [2]. The life cycles of these genera involve two hosts: a vertebrate and an arthropod. During a blood meal, the latter can transmit sporozoites into the vertebrate host, in which they can infect erythrocytes. However, malaria parasites first invade liver cells and then infect red blood cells (RBCs), in which they undergo asexual reproduction. After these stages, both *Babesia* and *Plasmodium* differentiate into male and female gametocytes. These are ingested by the arthropod host, in which the gametes unite and undergo a sporogonic cycle that results in the production of sporozoites. A new cycle begins when the arthropod host bites a new vertebrate host. These two intraerythrocytic protozoan parasite genera have such a similar life cycle, morphology and pathogenicity, and share such similar vertebrate and arthropod vectors, that they are often confused, although there are differences in their multiplication, egress and the roles of their parasitophorous vacuoles [3].

Interestingly, although artemisinin and its derivatives (ARTs) efficiently kill malaria parasites, they only slightly inhibit the growth of *Babesia* species [4–6]. Although both *Babesia* and *Plasmodium* digest hemoglobin for its amino acids, only *Plasmodium* spp. produce hemozoin in this metabolic process. Previously, hemozoin was widely considered an insoluble metabolic byproduct of hemoglobin digestion during the infection of RBCs by parasites, which lacked a biological function. However, there is increasing evidence that hemozoin plays a crucial role in the detoxification of free haem, and in the storage and utilization of iron [7–9]. Malaria parasites in particular always maintain a high haem level (1.6 µmol/L), far higher than that seen in other parasites, throughout their development in RBCs [10]. In addition, the accumulation of hemozoin in *Plasmodium* gametocytes suggests that hemozoin is of biological significance in the reproduction of species of this genus. Interestingly, malaria parasites that cannot synthesize hemozoin are similar to *Babesia* in their morphology and pathogenicity, and are also less virulent and have a lower rate of reproduction, e.g. as seen in some chloroquine-resistant *Plasmodium* spp., than malaria parasites that can synthesize this substance [11, 12].

It is not clear why *Babesia* do not produce hemozoin, a haem polymer that stores a large amount of HI. Malaria parasites are sensitive to iron chelators, which suggests that they depend on iron more than organisms that do not show this sensitivity. In particular, recent research indicates that artemisinin kills *Plasmodium* through an HI-use-disturbance effect [13]. It appears that *Plasmodium* and *Babesia* have different HI requirements, which may be of relevance to their different sensitivities to ARTs. Why, unlike *Plasmodium* spp., *Babesia* spp. do not require or store high amounts of HI and do not produce hemozoin, and whether the different requirements of these species for HI determine their different fecundities, have yet to be elucidated. To investigate the mechanisms underlying these differences, we compared the genomes of *P. yoelii* and *B. microti* and analyzed the effects of artemether on *P. yoelii* 17XNL and *B. microti* using single–cell transcriptomic sequencing, and examined the effect of supplying iron on the growth of *Babesia* in vivo.

## Methods

### Ethics statement

This study was carried out in strict accordance with the recommendations of the Regulations for the Administration of Affairs Concerning Experimental Animals of the State Science and Technology Commission. The protocol was approved by the Internal Review Board of Tongji University School of Medicine (TJLAC-017–039).

### Comparison between *Babesia* and *Plasmodium* genomes

To systematically explore the differences between the genomes of 53 *Plasmodium* and six *Babesia* species/strains (Additional file 1: Table S1), the *Plasmodium* Informatics Resource database (PlasmoDB), Piroplasma Informatics Resources database (Piroplasmadb), and Eukaryotic Pathogen, Vector and Host Informatics Resource (VEuPathDB) [14] were utilized to classify genes that have a specified orthology-based phylogenetic profile into three subgroups. To further explore the functions of these three categories of genes, Gene Ontology (GO) enrichment and metabolic pathway enrichment (using the algorithms from the KEGG pathway and Metabolic Pathways From all Domains of Life database) were carried out using the online tools in PlasmoDB (https://plasmodb.org/plasmo/app/workspace/strategies/), with a default* p*-value cutoff of 0.05. For the evaluation of the integrity of the haem biosynthesis (de novo) pathway, we retrieved information using ID EC00860 (KEGG annotation) in the PlasmoDB database (https://plasmodb.org/plasmo/app/record/pathway/KEGG/ec00860) and PriplasmaDB database (https://piroplasmadb.org/piro/app/record/pathway/KEGG/ec00860#drawing) [15].

### Experimental animals

*Plasmodium yoelii* 17XNL-EGFP was provided by Dr. Ana Rodriguez (New York University) and Dr. Wenyue Xu (Department of Pathogenic Biology, Army Medical University). *Babesia microti* strain ATCC®PRA-99TM was provided by the National Institute of Parasitic Diseases, Chinese Center for Disease Control and Prevention. *Plasmodium yoelii* 17XNL-EGFP was cultured in female BALB/c mice or ICR mice. *Babesia microti* was cultured in NOD/SCID mice. All of the mice were purchased from Shanghai Laboratory Animal Center (Shanghai, China) and housed in the Animal Center Laboratory of Tongji University. The NOD/SCID and BALB/c mice were inoculated with 200 μl of the cell suspension, i.e. 1–5 × 10^7^ *B. microti* or *P. yoelii* 17XNL-EGFP, by intraperitoneal injection. When the infection rate of *P. yoelii* 17XNL reached 25%-50%, artemether (100 mg/kg) was given to the mice by gavage. Blood was then sampled at 0 and at 24 h after artemether treatment.

### Cell isolation and cell sorting

At 0 and 24 h after artemether treatment, one or two drops of the blood sampled from the mice infected with *P. yoelii* 17XNL-EGFP parasites were collected in a 15-ml tube containing 10 ml of RPMI 1640 medium and immediately transferred on ice for cell sorting; the controls were treated similarly. The cell samples were sorted on a BD FACS-Aria II by using ultraviolet, blue, and red lasers at 355, 488, and 633 nm, respectively. After sorting, only infected erythrocytes were collected for the single-cell RNA-sequencing experiment, in accordance with a previous study [13]. One or two drops of the sampled blood from the mice infected with *B. microti* parasites were collected in 10 ml of RPMI 1640 media with 5% serum. These were then stained with CD45 antibody (1:200; catalogue no. 103,101; BioLegend) in the dark at room temperature for 20 min to identify leukocytes, and then incubated with Hoechst 33342 (1:100, catalogue no. c1028; Biyuntian) at room temperature for 10 min. The control group did not undergo any treatment. The samples were washed and resuspended in RPMI 1640 medium before FACS sorting. A BD FACS Aria II was used to obtain a sufficient number of infected RBCs for single-cell sequencing. Positive cells were collected in RPMI 1640 medium supplemented with 5% serum. The cells were stained with 0.4% trypan blue to check their viability by using an automated cell counter. The cell samples were sorted on a BD FACSAria II by using ultraviolet, blue and red lasers at 355, 488, and 633 nm, respectively. After sorting, only infected erythrocytes were collected for the single-cell RNA-sequencing experiment.

### Chromium 10x Genomics library and sequencing

Single-cell suspensions were loaded onto 10x Chromium to capture approximately 3000–10,000 single cells, in accordance with the manufacturer’s instructions for the 10x Genomics Chromium Single-Cell 3’ Kit. The complementary DNA amplification and library construction steps were performed in accordance with the standard protocol. The libraries were sequenced on an Illumina NovaSeq 6000 sequencing system (paired-end multiplexing run, 150 base pairs; Majorbio, Shanghai, China).

### Single-cell RNA sequencing

The reads were processed using the Cell Ranger 4.0 pipeline with default and recommended parameters. FASTQs generated from the Illumina sequencing output were aligned to the *Plasmodium* genomes (GenBank assembly GCA_900002385.2) and *Babesia* genomes (assembly ASM69194v2) by using the STAR algorithm [16]. Gene-barcode matrices were then generated for each individual sample by counting unique molecular identifiers and filtering non-cell-associated barcodes. Finally, a gene-barcode matrix containing the barcoded cells and gene expression counts was generated. This output was then imported into the Seurat (v3.2.0) R toolkit for quality control and downstream analysis of the single cell RNA-sequencing data [17]. All functions were run with default parameters unless specified otherwise. Low-quality cells were filtered using a standard panel of three quality criteria: number of detected transcripts (number of unique molecular identifiers), detected genes, and percent reads mapping to mitochondrial genes (quartile threshold screening criteria). The normalized data (NormalizeData function in the Seuratpackage) were used to extract a subset of variable genes. Variable genes were identified while controlling for the strong relationship between variability and average expression.

### Identification of cell types and subtypes by dimensional reduction and cluster analysis

Gene expression data for each voxel were normalized by using sctransform [18] in Seurat, which uses regularized negative binomial models to account for technical artifacts while preserving biological variance. Then, the top 30 principal components were calculated and used to construct a KNN graph. The Louvain algorithm was used to cluster the voxels. We visualized the clusters on a two-dimensional map produced with Uniform Manifold Approximation and Projection (UMAP). For each cluster, we used the Wilcoxon rank-sum test to find significant deferentially expressed genes by comparing the remaining clusters.

### Differential expression analysis and functional enrichment

The differentially expressed genes (DEGs) between two different samples or clusters were obtained using the function FindMarkers in Seurat, using a likelihood ratio test. Essentially, |log2FC| > 0.25 and *Q* <  = 0.05 were considered to indicate genes that were significantly differentially expressed. In addition, GO functional enrichment analysis was performed to identify which DEGs were significantly enriched in GO terms and metabolic pathways at a Bonferroni-corrected *P*-value of ≤ 0.05 compared with the whole transcriptome background. GO functional enrichment analyses were carried out by using GOATOOLS (https://github.com/tanghaibao/Goatools).

### RNA velocity analysis

RNA velocity was calculated based on spliced and unspliced counts, as previously reported [19], and cells that were present in the pseudotemporal ordering were used for the analysis. We estimated the RNA velocity by scVelo (https://scvelo.org) [20], a method of developmental trajectory analysis. It estimates the variation of RNA abundance over time by calculating the ratio of messenger RNA before and after splicing in cells, and infers the next possible differentiation direction of cells. To plot individual cell velocities, the UMAP and T-SNE embeddings in Seurat were exported.

### In vivo iron dextran assay

In the in vivo experiment, 45 BALB/c mice were infected with (1–10) × 10^6^ *B. microti*-parasitized erythrocytes by intraperitoneal injection. Then, they were randomly divided into three groups, 1, 2 and 3. From the second day of infection on, group 1 mice were administered a subcutaneous injection of 1 g/kg iron dextran every 2 days and group 2 mice were administered a subcutaneous injection of 1.25 g/kg iron dextran every 2 days. By contrast, group 3 mice were injected subcutaneously with an equivalent amount of saline. Blood samples were then collected from the tail veins of the mice once daily to examine the infection rates by Giemsa staining.

### Tissue preparation and immunofluorescence assay

The spleens were removed from the mice 12 days after infection with *B. microti*, and were fixed in a 10% neutral paraformaldehyde fix solution. For immunofluorescence staining, the paraffin sections of the mice spleens were deparaffinized and rehydrated in ethanol and rinsed in phosphate buffered saline. The sections were first incubated with CD206 antibody (1:2000, GB113497) and then covered with horseradish peroxidase-labeled goat anti-rabbit immunoglobulin G (1:500, GB23303) at room temperature for 50 min in the dark. The slides were washed three times with phosphate buffered saline (pH 7.4) and then incubated with TSA-FITC solution for 10 min. After the primary antibodies and secondary antibodies had been removed, the slides were incubated with anti-mouse inducible nitric oxide synthase (iNOS) antibody (1:200, GB11119) and the second corresponding secondary antibody, CY3-goat anti-rabbit immunoglobulin G (1: 300, GB21303). Finally, the sections were incubated with 4′,6-diamidino-2-phenylindole (G1012) for 10 min at room temperature.

### Serum cytokine analysis

Mice were sacrificed 12 days after infection with *B. microti* and blood and spleen samples collected. Multiplex kits for measuring cytokines were purchased from Bio-Rad (Bio-Plex Pro Mouse Cytokine Grp I Panel 23-plex). Cytokine analyses were performed by Wayen Biotechnologies (Shanghai, China) using the Bio-Plex MagPix System (Luminex, Austin, TX) following the manufacturer’s instructions for Luminex xMAP technology with multiplex beads. Bio-Plex Manager version 6.1 software (Luminex) was used to calculate the cytokine concentrations of the uninfected normal group, the infected group without iron dextran treatment, and the infected group with 1.25 g/kg of iron dextran treatment. A nonlinear least-squares minimization algorithm generated a curve fitted by a five-parameter logistic equation and determined the high and low limits of detection. Twenty-three cytokines were measured.

### Statistical analysis

Graphpad Prism 8.0.2 software was used to perform the statistical analysis. Student’s* t*-test (unpaired) was performed to compare the changes in parasitemia in the groups in the in vivo iron dextran assay. A chi-square test of independence was used to compare changes in the number of parasites expressing a special gene in different populations after artemether treatment. A *P*-value of < 0.05 was considered to indicate statistical significance.

## Results

### *Babesia* genomes lack enzymes of the complete haem synthesis system as compared to *Plasmodium* genomes

**Fig. 1:**
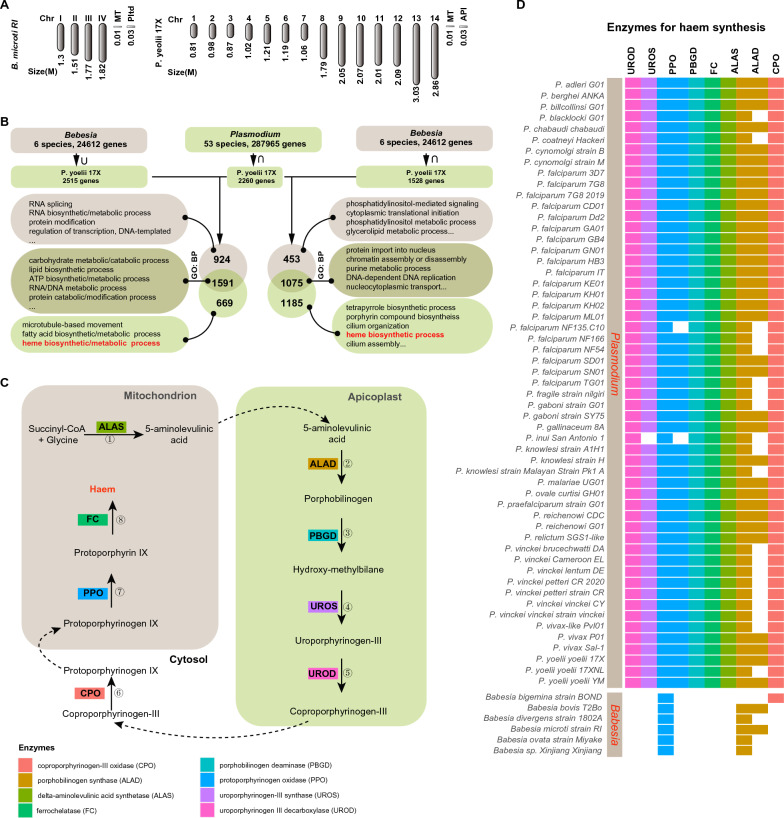
Comparison of *Babesia* and *Plasmodium* genomes and the distribution of haem synthesis-related genes in them. **a** Schematic of *Babesia microti* and *Plasmodium yoelii* chromosomes. **b** Schematic of the analysis of the common and special genes of *B. microti* and *P. yoelii*. **c** Schematic of the process of haem synthesis and the involvement of eight key enzymes. **d** Distribution of haem synthesis-related genes in the *Babesia* and *Plasmodium* genomes

*Babesia* genomes comprise four chromosomes, and range in size from 6 to 15 Mb [e.g. *Babesia microti* (6.44 Mb), *Babesia bovis* (8.18 Mb), *Babesia bigemina* (12.84 Mb), *Babesia ovata* (14.45 Mb), *Babesia ovis* (8.38 Mb) and *Babesia divergens* (9.65 Mb) (Additional file 1: Table S1; Fig. 1a)]. By contrast, *Plasmodium* genomes comprise 14 chromosomes, and range in size from 14 to 38 Mb [e.g. *Plasmodium falciparum* (23.49 Mb), *Plasmodium vivax* (29.04 Mb), *Plasmodium yoelii* (22.45 Mb), and *Plasmodium malariae* (31.92 Mb) (Additional file 1: Table S1; Fig. 1a)]. To investigate the commonality and the individuality of *Babesia* and *Plasmodium*, we systematically explored the differences between the genomes of 53 *Plasmodium* and six *Babesia* species/strains (Fig. 1b). PlasmoDB, a well-known *Plasmodium* informatics resource [14], was utilized to classify genes that have a specific orthology-based distribution profile into three subgroups: 669 *Plasmodium*-specific genes (Additional file 2: Table S2), 924 *Babesia*-specific genes (Additional file 3: Table S3), and 1591 genes shared by *Plasmodium* and *Babesia* (Additional file 4: Table S4). GO analysis highlighted three terms: microtubule-based movement, fatty acid biosynthetic/metabolic process, and haem biosynthetic/metabolic process (Fig. 1b). To determine the differences between their core functions, we compared their intersections, and thus obtained another three subgroups: 1185 *Plasmodium*-specific genes (Additional file 5: Table S5), 453 *Babesia*-specific genes (Additional file 6: Table S6), and 1075 genes shared by *Plasmodium* and *Babesia* (Additional file 7: Table S7). Of note, some biological processes are only indicated in the *Plasmodium*-specific genes group, such as a porphyrin-containing compound biosynthetic process, a haem biosynthetic process, a porphyrin-containing compound metabolic process, a tetrapyrrole biosynthetic process, a tetrapyrrole metabolic process, a protoporphyrinogen IX metabolic process, a protoporphyrinogen IX biosynthetic process, a haem metabolic process, a pigment biosynthetic process, and a pigment metabolic process (Additional file 1: Table S2; Additional file 5: Table S5). In particular, haem-synthesis genes widely exist among various organisms and play a vital role in life processes. We analyzed haem synthesis genes in *Plasmodium* and *Babesia* genomes.

Haem is biologically synthesized by a complex enzymatic reaction mediated by eight enzymes, namely coproporphyrinogen-III oxidase, delta-aminolevulinic acid dehydratase, delta-aminolevulinic acid synthase, ferrochelatase, porphobilinogen deaminase, protoporphyrinogen oxidase, uroporphyrinogen III decarboxylase, and uroporphyrinogen III synthase (Fig. 1c) [21]. To further evaluate the significance of haem metabolism, we analyzed the integrity of the haem biosynthesis pathway in 53 *Plasmodium* and six *Babesia* species/strains in detail by using VEuPathDB and KEGG. VEuPathDB showed that approximately 92.3% (51/53) of these *Plasmodium* strains have an integrated enzymatic system for the de novo synthesis of haem, and that *Plasmodium inui* San Antonio 1 and *Plasmodium falciparum* NF135.C10 do not (Fig. 1d). KEGG revealed that several of the *Plasmodium* species lack a complete enzyme system for the de novo synthesis of haem (Additional file 8: Table S8). However, only protoporphyrinogen oxidase and delta-aminolevulinic acid dehydratase were identified in the genomes of the six *Babesia* species (Fig. 1d), suggesting that not all *Babesia* have the ability to biologically synthesize haem de novo.

### Key enzyme genes in the pentose phosphate pathway in *Babesia* are not as actively expressed as those in the pentose phosphate pathway in *Plasmodium*

**Fig. 2:**
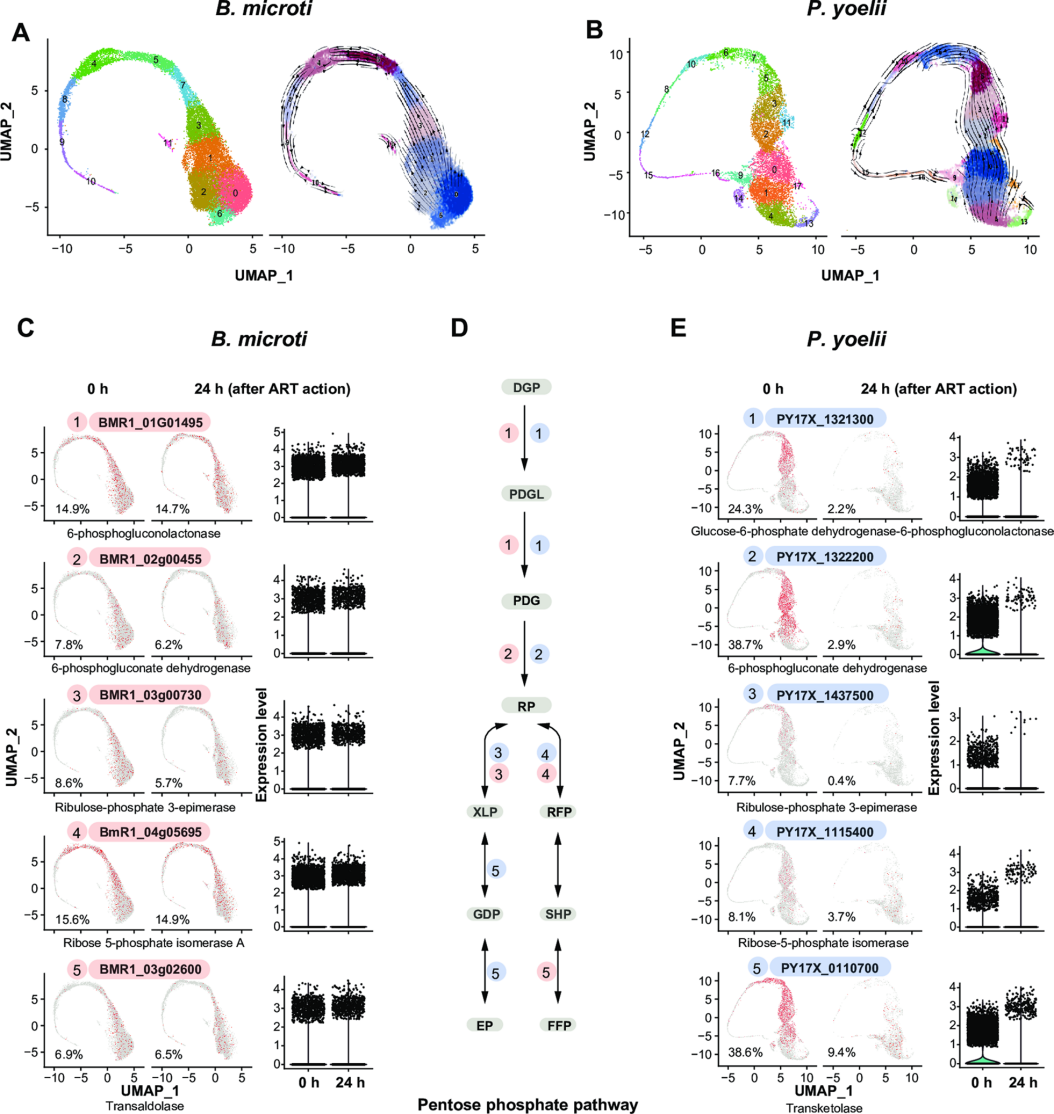
Uniform Manifold Approximation and Projection (UMAP) plots showing the expression characteristics of key enzyme genes in the pentose phosphate pathway (PPP) in *Babesia microti* and *Plasmodium yoelii* 17XNL, and the sensitivity of the parasites that expressed these genes to artemether (ART). **a** UMAP plot of single-cell transcriptomes (SCTs) from the artemether-treated and control groups of *B. microti.* RNA velocity analysis showing the developmental relationships among different clusters of *B. microti.* **b** UMAP plot of SCTs from the artemether-treated and control groups of *P. yoelii* 17XNL*.* RNA velocity analysis showing the developmental relationships between different clusters of *P. yoelii* 17XNL.** c** UMAP plots showing the expression characteristics of 6-phosphogluconolactonase, 6-phosphogluconate dehydrogenase, ribulose-phosphate 3-epimerase, ribose 5-phosphate isomerase A, and transaldolase, and the changes in the percentages of parasites expressing these genes in the population after 24 h of artemether treatment. **d** Schematic of PPP. **e** UMAP plots showing the expression characteristics of glucose-6-phosphate dehydrogenase-6-phosphogluconolactonase, 6-phosphogluconate dehydrogenase, ribulose-phosphate 3-epimerase, ribose-5-phosphate isomerase, and transketolase, and changes in the percentages of parasites expressing these genes in the population after 24 h of artemether treatment. Chi-square test was used to test the statistical significance of differences between the percentages of parasites expressing the same gene in the *B. microti* group and the *P. yoelii* 17XNL groups 24 h after ART treatment.* P* < 0.001 was obtained for all comparisons.* DGP* d-glucopyranose 6-phosphate,* PDGL* 6-O-phosphonato-d-glucono-1,5-lactone,* PDG* 6-phosphonatooxy-D-gluconate,* RP* d-ribulose 5-phosphate, XLP d-xylulose 5-phosphate,* RFP* d-ribofuranose 5-phosphate,* GDP* d-glyceraldehyde 3-phosphate,* SHP* sedoheptulose 7-phosphate,* EP* d-erythrose 4-phosphate,* FFP* beta-d-fructofuranose 6-phosphate

In malaria parasites, hemoglobin is degraded to release haem. HI play a crucial role in linking hemoglobin degradation with the pentose phosphate pathway (PPP) which continuously produces nicotinamide adenine dinucleotide phosphate for the reduction of oxidized glutathione and thioredoxin and ribose-5-phosphate for nucleotide biosynthesis. This cycle is called the hemoglobin-haem-iron-pentose phosphate pathway (HHIP) [13]. When the HHIP is sustained, ribose-5-phosphate can be continuously supplied for DNA synthesis [13]. We found that both *B. microti* and *P. yoelii* 17XNL have all the PPP-related enzymes (Fig. 2). Based on the distribution in the UMAP plots of the parasites that expressed PPP-related genes, we found that the percentage of *B. microti* expressing these genes in a population was not as high as in *P. yoelii* 17XNL. The former ranged from 6.9% to 15.6% (Fig. 2c) while the latter ranged from 7.7% to 38.7% (Fig. 2e). In particular, far higher percentages of *P. yoelii* 17XNL expressed glucose-6-phosphate dehydrogenase-6-phosphogluconolactonase (PY17X_1321300; 24.3%), 6-phosphogluconate dehydrogenase (PY17X_1322200; 38.7%) and transketolase (PY17X_0110700; 38.6%) than did *B. microti* (Fig. 2c–e). Within a population, the percentage of malaria parasites that expressed these genes was higher than the percentage of *B. microti* that expressed them. It is possible that these genes are more actively expressed in malaria parasites.

### *Babesia microti* that express PPP—related genes are not susceptible to artemether

The percentages of *B. microti* that expressed key genes of the PPP, such as 6-phosphogluconolactonase (BMR1_01G01495), 6-phosphogluconate dehydrogenase (BMR1_02g00455), ribulose-phosphate 3-epimerase (BMR1_03g00730), ribose 5-phosphate isomerase A (BmR1_04g05695), and transaldolase (BMR1_03g02600), in this population had hardly changed 24 h post-artemether treatment (Fig. 2c, d). For example, the percentage of *B. microti* that expressed 6-phosphogluconolactonase only decreased from 14.9% to 14.7% after 24 h of artemether treatment. By contrast, the percentage of *P. yoelii* decreased from 24.3% to 2.2% (Fig. 2c, d). Similarly, the percentage of *B. microti* that expressed 6-phosphogluconate dehydrogenase only decreased from 7.8% to 6.2%, whereas the percentage of *P. yoelii* decreased from 38.7% to 2.9%. It was clear that more malaria parasites expressing glucose-6-phosphate dehydrogenase-6-phosphogluconolactonase (PY17X_1321300), 6-phosphogluconate dehydrogenase, decarboxylating (PY17X_1322200), ribulose-phosphate 3-epimerase (PY17X_1437500), ribose-5-phosphate isomerase (PY17X_1115400), and transketolase (PY17X_0110700) were eliminated 24 h post-artemether treatment than *B. microti* (Fig. 2e).

### *Babesia microti* that express DNA synthesis-, antioxidation-, and glycolysis-related genes are not susceptible to artemether

**Fig. 3:**
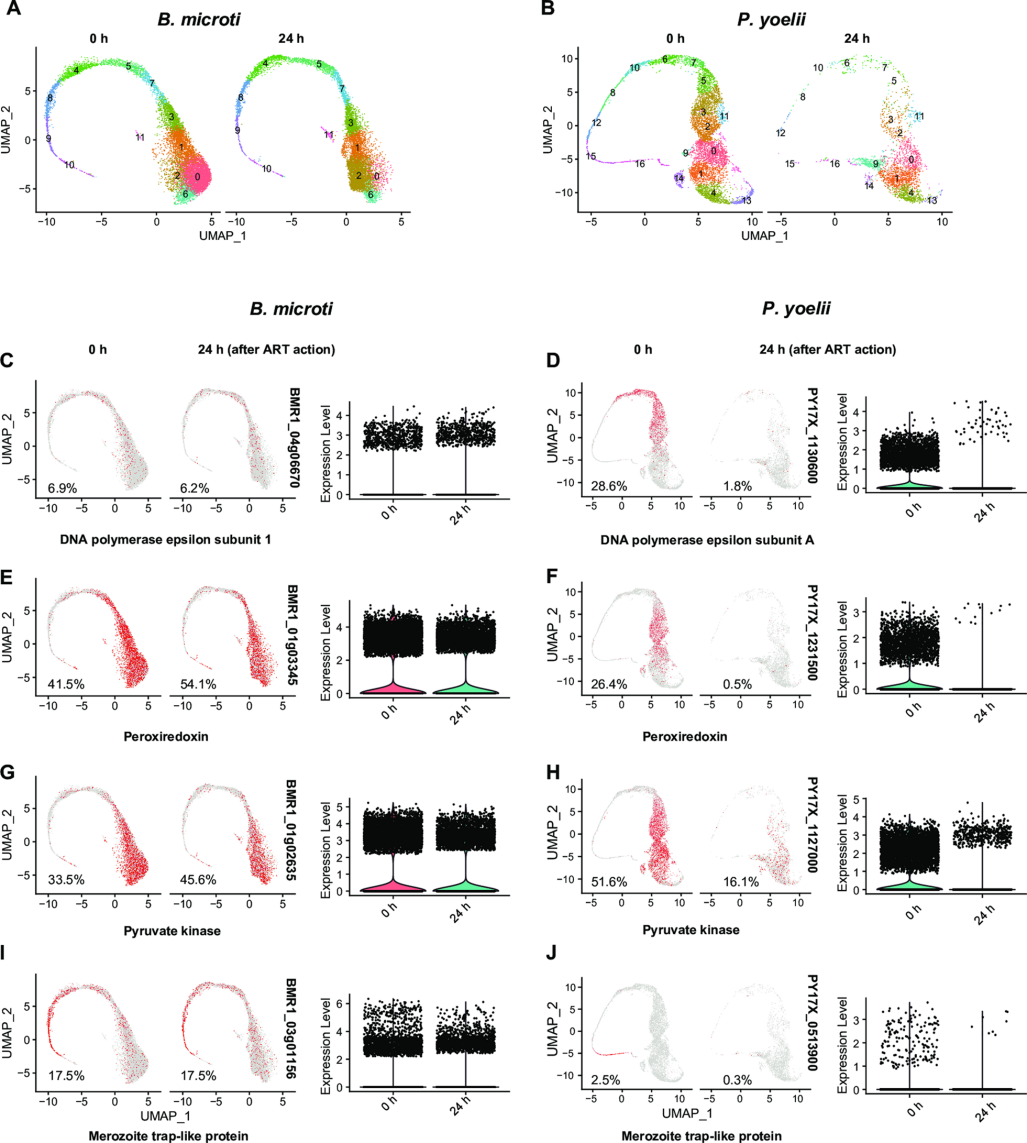
UMAP plots showing the sensitivity of *Babesia microti* and *Plasmodium yoelii* 17XNL that expressed different genes at 24 h of artemether treatment. **a** UMAP plot of single-cell transcriptomes (SCTs) from the artemether-treated and control groups of *B. microti*, showing the sensitivity of *B. microti* to 24 h of artemether treatment. **b** UMAP plot of SCTs from the artemether-treated and control groups of *P. yoelii* 17XNL, showing the sensitivity of *P. yoelii* 17XNL to 24 h of artemether treatment. **c** UMAP plots showing the expression characteristics of DNA polymerase epsilon subunit 1 (BMR1_04g06670) and the change in the percentage of parasites expressing the gene in the population after 24 h of artemether treatment. **d** DNA polymerase epsilon subunit A (PY17X_1130600). **e** Peroxiredoxin (BMR1_01g03345). **f** Peroxiredoxin (PY17X_1231500). **g** Pyruvate kinase (BMR1_01g02635). **h** Pyruvate kinase (PY17X_1127000). **i** Merozoite trap-like protein (BMR1_03g01156). **j** Merozoite trap-like protein (PY17X_0513900). *P* < 0.001 was obtained for all comparisons

The percentage of *B. microti* expressing DNA polymerase epsilon catalytic subunit 1 was 6.9% in one of the treatment groups. After 24 h of artemether treatment, the percentage had dropped to 6.2%. By contrast, the percentage of *P. yoelii* 17XNL expressing the same enzyme was 28.6%, and the percentage dropped to 1.8% after 24 h of artemether treatment (Fig. 3c, d; Additional file 9: Fig. S1). Similar results were observed in the other *B. microti* groups which expressed antioxidation-related genes, such as peroxiredoxin and thioredoxin reductase, and glycolysis-related genes, such as pyruvate kinase and hexokinase (Fig. 3c–h). For example, 41.5% of the parasites expressed the peroxiredoxin gene in the *B. microti* group. After 24 h of artemether treatment, the percentage had increased to 54.1%. Meanwhile, the percentage of *P. yoelii* 17XNL expressing the same gene dropped from 26.4% to 0.5% after 24 h of artemether treatment (Fig. 3e, f). It was clear that *P. yoelii* 17XNL that expressed these genes were eliminated by artemether, while the *B. microti* were not.

In addition, at a later stage, *B. microti* that expressed merozoite trap-like protein and apical merozoite protein genes were not sensitive to artemether either, unlike *P. yoelii* 17XNL (Fig. 3i, j). These results indicate that, in contrast to *P. yoelii* 17XNL, nearly all of the stages of *B. microti* showed no susceptibilty to artemether with respect to their DNA synthesis, antioxidation, glycolysis, and reproduction (Fig. 3; Additional file 10: Fig. S2).

### Glutathione-related genes and the superoxide dismutase [Fe] gene of *B. microti* are not as actively expressed as those of *P. yoelii* 17XNL

**Fig. 4:**
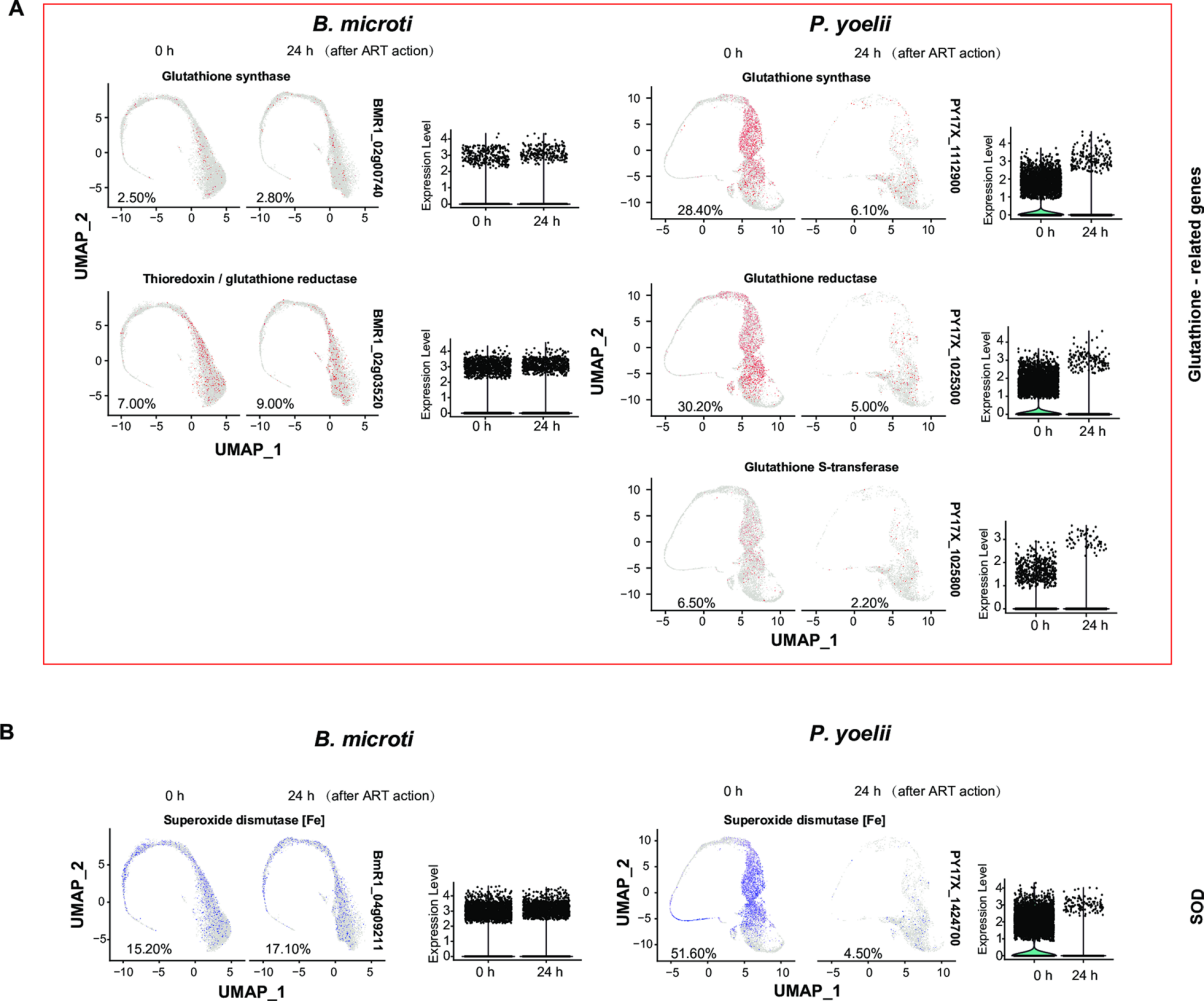
UMAP plots showing the differences in the numbers and gene expression levels of *B. microti* and *Plasmodium yoelii* 17XNL that expressed glutathione-related genes and the superoxide dismutase gene. **a** UMAP plots showing the differences in the numbers and gene expression levels and artemether sensitivity of *B. microti* and *P. yoelii* 17XNL that expressed glutathione-related genes. Most of the *P. yoelii* 17XNL that expressed these genes had been eliminated at 24 h of artemether treatment, whereas the *B. microti* were hardly affected by the same treatment. **d** Differences in the numbers and gene expression levels and artemether sensitivity of *B. microti* and *P. yoelii* 17XNL that expressed the superoxide dismutase [Fe] gene.* P* < 0.001 for all comparisons

In malaria parasites, hemoglobin is ingested and degraded, releasing haem. Haem is degraded by glutathione into iron and superoxide [22]. Superoxide dismutase [Fe] (SOD) can catalyze the dismutation of superoxide to hydrogen peroxide. Of note, in the HHIP of malaria parasites, the degradation of haem by glutathione to iron is a key step [13]. The released iron activates PPP [22] to continuously produce nicotinamide adenine dinucleotide phosphate and ribose-5-phosphate for nucleotide biosynthesis. Fewer *B. microti* than *P. yoelii* 17XNL expressed glutathione synthase and thioredoxin/glutathione reductase genes. Moreover, the genome of *B. microti*, unlike that of *P. yoelii* 17XNL, does not contain the glutathione S-transferase gene (Fig. 4a). In addition, expression of the SOD gene is seen in nearly all stages of of *B. microti* rather than being concentrated in specific stages, as in *P. yoelii* 17XNL (Fig. 4b). Furthermore, the number of *B. microti* that expressed the SOD gene at earlier stages was far lower than the number of *P. yoelii* 17XNL. Most of the *P. yoelii* 17XNL that expressed these genes had been eliminated at 24 h of artemether treatment. By contrast, *B. microti* were hardly affected by the same treatment (Fig. 4a, b).

### Supplying iron promotes the reproduction of *B. microti*

**Fig. 5:**
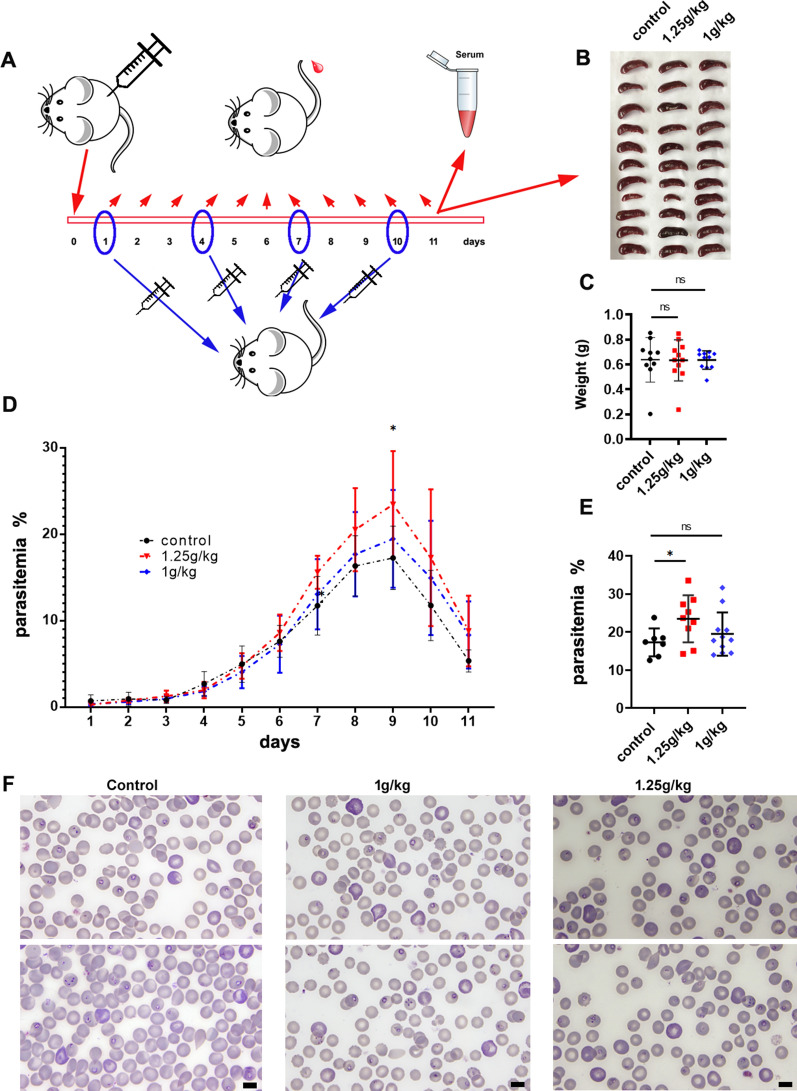
The effect of supplying iron on the in vivo infection rate of mice with *B. microti*. **a** Schematic of the experimental process, produced using open source alternatives (https://openclipart.org/). **b** Spleen samples from the infected mice in the control, 1 g/kg, and 1.25 g/kg test groups. **c** Comparison of the weights of the sampled spleens. **d** The effect of supplying iron on parasitemia due to *B. microti*. **e** Comparison of the infection rates of *B. microti* on day 9. **f** Blood smears showing the morphology of *B. microti* and different levels of infection in the three groups on day 9. Scale bars indicate 5 μm. * *P* < 0.05, *ns* not significant (*P* > 0.05)

Parasitemia in the mice generally peaked 8–10 days post-infection with *B. microti*. When the mice were injected with iron dextran, the infection rate gradually increased with time and dosage (Fig. 5a, d, e). Parasitemia peaked 9 days post-infection. Parasitemia was higher in the experimental group than in the control group. Parasitemia was significantly higher in the group that was administered subcutaneous injections of 1.25 g/kg iron dextran compared to the other groups (Fig. 5a, d–f). All of the groups were infected by *B. microti*, and enlarged spleens were observed as a consequence of infection (Fig. 5b). There was no significant difference in spleen weights, but the spleens were darker in color in the experimental groups, and especially in the group treated with 1.25 g/kg iron dextran (Fig. 5c).

### Supplying iron did not change macrophage M1/M2 polarization in mice infected with *B. microti*

**Fig. 6:**
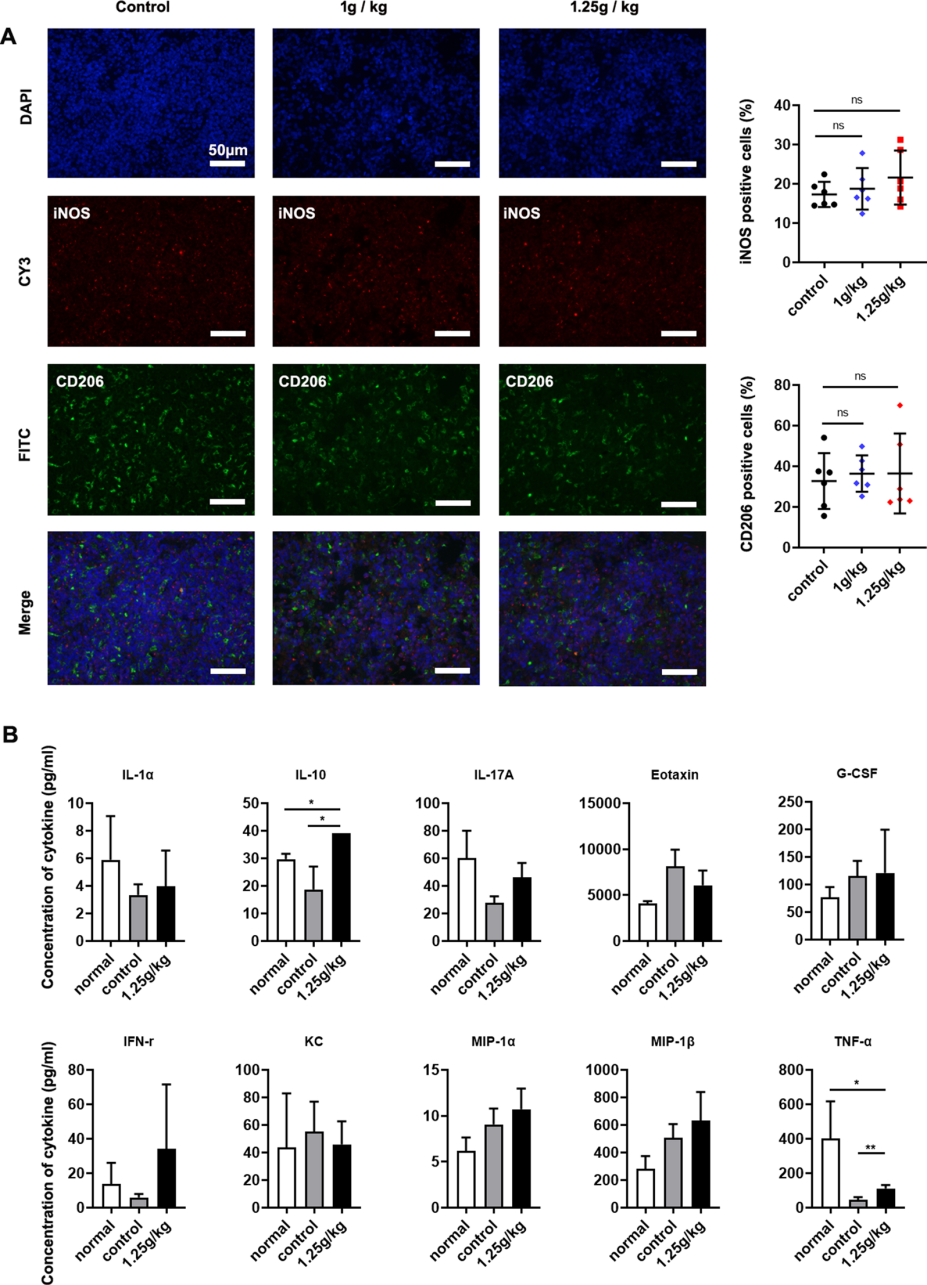
Effect of supplying iron to *B. microti*-infected mice on macrophage and cytokine secretion. **a** Immunofluorescence staining was used to analyze the expression of M1 and M2 macrophage-specific markers, inducible nitric oxide synthase (iNOS) and CD206, in mouse spleen tissue sections (scale bar = 50 µm;* n* = 6 per group). Control group, no iron supplied to infected mice; 1 g/kg group, subcutaneous injection of 1 g/kg iron dextran into infected mice every 2 days; 1.25 g/kg group, subcutaneous injection of 1.25 g/kg iron dextran into infected mice every 2 days.** b** Expression of interleukin (IL)-1α, IL-10, IL-17A, eotaxin, G-CSF, IFN-γ, KC, MIP-1α MIP-1β, and TNF-α in mice sera was detected by using Bio-Plex Pro Mouse Cytokine Grp I Panel 23-plex (*n* = 6 per group). Normal group, uninfected group; control group, infected mice without iron dextran injection; 1.25 g/kg group, infected mice with subcutaneous injections of 1.25 g/kg iron dextran every 2 days. * *P* < 0.05, ** *P* < 0.01, *ns* not significant (*P* > 0.05)

We identified the phenotypes and numbers of macrophages using immunohistochemistry and fluorescence in situ hybridization in mouse spleen specimens. Changes in the fluorescence intensity of fluorescent antibodies of iNOS and CD206 showed that the number of macrophages positive for iNOS and CD206 was similar for all test groups and control groups. No significant changes were found between the groups (Fig. 6a).

### Cytokine analysis

Cytokines in mouse sera were evaluated by Bio-Plex Pro mouse cytokine 23-plex assays. Significant differences were identified for interleukin (IL)-10 and TNF-α (Fig. 6b). Levels of other cytokines, including IL-1α, IL-17A, eotaxin, G-CSF, INF-γ, KC, MIP-1α, MIP-1α, MIP-1β, and TNF-α, did not significantly differ between the three groups. According to these results, iron dextran does not affect the production of most cytokines in vivo. Although iron can stimulate the production of the anti-inflammatory IL-10, it also stimulates TNF-α production.

## Discussion

*Babesia* infect erythrocytes and likely digest hemoglobin to utilize the products of its degradation [3], although this process is not as yet well understood. Clearly, *Babesia* are not sensitive to ARTs, but malaria parasites are. Moreover, malaria parasites are sensitive to iron chelators [23–28], whereas *Babesia* are not [29]. In particular, malaria parasites can produce and store hemozoin and sustain a high haem level throughout their development in RBCs [10]. Furthermore, *Plasmodium* genomes comprise all the genes necessary for haem synthesis, whereas *Babesia* genomes do not. It appears that *Babesia* do not rely on haem or iron as much as malaria parasites do. Given that ARTs require haem or iron for their activation, the requirement for HI likely determines sensitivity to ARTs. It was recently proposed that there is a double-kill mechanism of artemisinin against *Plasmodium* through HI-use-disturbance effect and free-radical effects [13]. In that study it was further suggested that ARTs kill parasites through their interaction with HI [13]. It is likely that the more a parasite requires, stores, utilizes, or relies on HI, the more it is sensitive to ARTs.

This raises the question of why malaria parasites require far more HI than *Babesia* do. Of note, DNA replication-related genes are expressed more actively in *P. yoelii* 17XNL than in *B. microti*. Moreover, both parasites have the complete PPP enzyme system, but more *P. yoelii* 17XNL showed high expression of DNA replication-related genes. In particular, *P. yoelii* 17XNL have an HHIP that continuously provides ribose-5-phosphate for nucleotide biosynthesis [13]. If *B. microti* only requires amino acids rather than haem after the digestion of hemoglobin, it likely lacks the HHIP. Of note, genes of relevance to PPP and glutathione-related genes in *B. microti* are inactively expressed. Even though the HHIP is present in *B. microti*, its efficiency is lower in this species than in *P. yoelii* 17XNL. Clearly, when the HHIP is sustained, ribose-5-phosphate can be continuously acquired for DNA synthesis, which undoubtedly facilitates the production of numerous merozoites. This is supported by the fact that *P. falciparum* can produce between eight and 32 merozoites, whereas *Babesia* can only produce between two and four. It appears that HI plays a crucial role in the development and reproduction of malaria parasites. Given that malaria parasites sustain a high haem level at nearly all of their stages in RBCs [10], it seems that all of these require haem, which likely explains why most of the stages were sensitive to 24 h of artemether treatment. By contrast, nearly all of the *B. microti* that expressed DNA synthesis-, antioxidation-, glycolysis-, reproduction- and glutathione-related genes were not susceptible to artemether. It is likely that HI dependence is the weak point in malaria parasites under artemisinin treatment, whereas the lack of this dependence seems to favor *Babesia*. Of note, ARTs can inhibit the growth of* Babesia* species [4–6], but their effective concentrations are much higher for these species than for* Plasmodium* spp. [5, 30–32]. It would be interesting to explore whether there is a mechanism common to both *Babesia* and* Plasmodium* which explains their sensitivity to artemisinin.

To investigate the significance of HI for *Babesia*, we supplied iron through subcutaneous injections of iron dextran in in vivo experiments. The infection rate of *Babesia* increased in a dose-dependent manner. Of note, the increase in the rate of infection with *B. microti* was not related to a change in macrophage polarization or cytokine secretion. It appears that the extra iron is beneficial as it promotes the reproduction of *B. microti*. This leads us to raise the question: why have *Babesia* not evolved similar mechanisms to malaria parasites for the utilization of HI to enhance their fertility?

## Conclusions

Compared to malaria parasites, which mostly have a genome that exceeds 20 Mb in size [33, 34], *Babesia* spp. have a genome of only about 6 Mb in size [35–37]. Of note, *Babesia* genomes do not include a complete set of genes for haem synthesis. Thus it is likely that *Babesia* spp. do not possess sufficiently large genomes to evolve similar mechanisms to those seen in malaria parasites for the accumulation and utilization of HI, which results in the lower fertility of the former. However, the fact that *Babesia* have no major requirement for HI, do not need to store a large amount of HI, and are not dependent on HI for many of their developmental stages, renders them insusceptible to ARTs treatments.

## Supplementary Information

**Additional file 1: Table S1.** Comparison of *Babesia* and *Plasmodium* genomes.

**Additional file 2: Table S2.** GO analysis of 669 *Plasmodium*-specific genes.

**Additional file 3: Table S3.** GO analysis of 924 *Babesia*-specific genes.

**Additional file 4: Table S4.** GO analysis of 1591 genes shared by *Plasmodium* and *Babesia.*

**Additional file 5: Table S5.** GO analysis of 1185 *Plasmodium*-specific genes.

**Additional file 6: Table S6.** GO analysis of 453 *Babesia*-specific genes.

**Additional file 7: Table S7.** GO analysis of 1075 genes shared by *Plasmodium* and *Babesia*.

**Additional file 8: Table S8.** OrthoMCL identifiersand haem biosynthesis enzymes.

**Additional file 9: Fig. S1.** Effects of 24 h of artemether treatment on *Babesia microti* and *Plasmodium yoelii* 17XNL that expressed DNA polymerases. The UMAP plots show that artemether did not affect *B. microti* that express DNA polymerase genes but did eliminate *P. yoelii* 17XNL that expressed similar genes.* ART* Artemether.

**Additional file 10: Fig. S2.** UMAP plots showing the sensitivity of *Babesia microti* and *Plasmodium yoelii* 17XNL that expressed different genes to 24 h of artemether treatment. **a** UMAP plots showing the expression characteristic of DNA polymerase alpha subunit Aand the change in the percentage of parasites expressing the gene in the population after 24 h of artemether treatment.** b** DNA polymerase alpha subunit A.** c** Thioredoxin reductase.** d** Thioredoxin reductase.** e** Hexokinase.** f** Hexokinase.** g** Merozoite trap-like protein.** h** Apical merozoite protein.* P* < 0.001 for all comparisons.

## Data Availability

All data generated or analyzed during this study are included in the article.

## Abbreviations

ARTs: Artemisinin and its derivatives
DEGs: Differentially expressed genes
GO: Gene Ontology
HHIP: Hemoglobin-haem-iron pentose phosphate pathway
HI: Haem and iron
iNOS: Inducible nitric oxide synthase
PPP: Pentose phosphate pathway
RBCs: Red blood cells
SOD: Superoxide dismutase
UMAP: Uniform Manifold Approximation and Projection
HHIP: Hemoglobin-haem-iron-pentose phosphate pathway
