# Supplementary figures and images for: Why is Babesia not killed by artemisinin like Plasmodium?
Source: Parasit Vectors. 2023 Jun 8;16:193. doi: 10.1186/s13071-023-05783-4 (PMC10249562; doi:10.1186/s13071-023-05783-4)

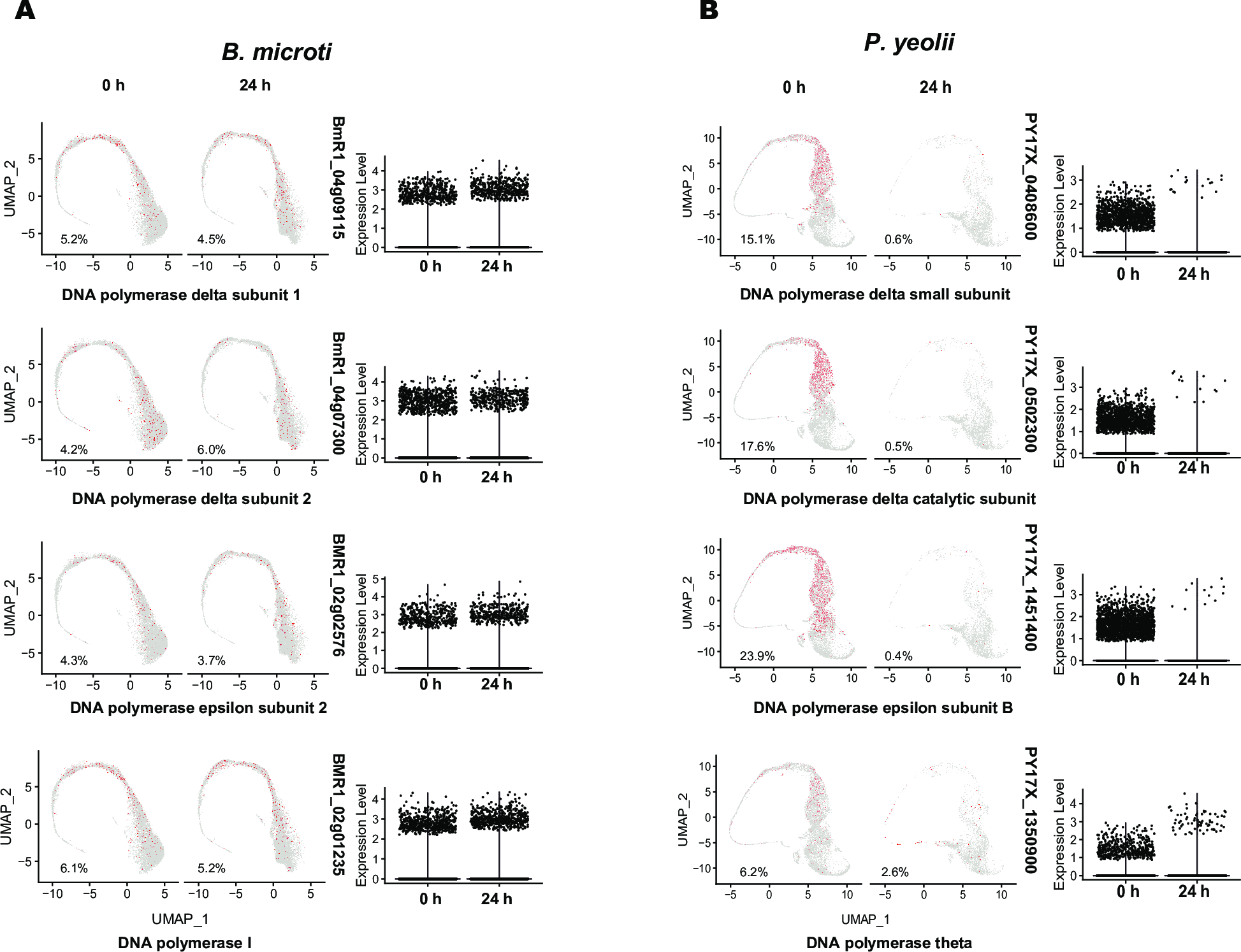

Supplement: Supplementary file 9 — Additional file 9: Fig. S1. Effects of 24 h of artemether treatment on Babesia microti and Plasmodium yoelii 17XNL that expressed DNA polymerases. The UMAP plots show that artemether did not affect B. microti that express DNA polymerase genes but did eliminate P. yoelii 17XNL that expressed similar genes. ART Artemether. [file 13071_2023_5783_MOESM9_ESM.tif]

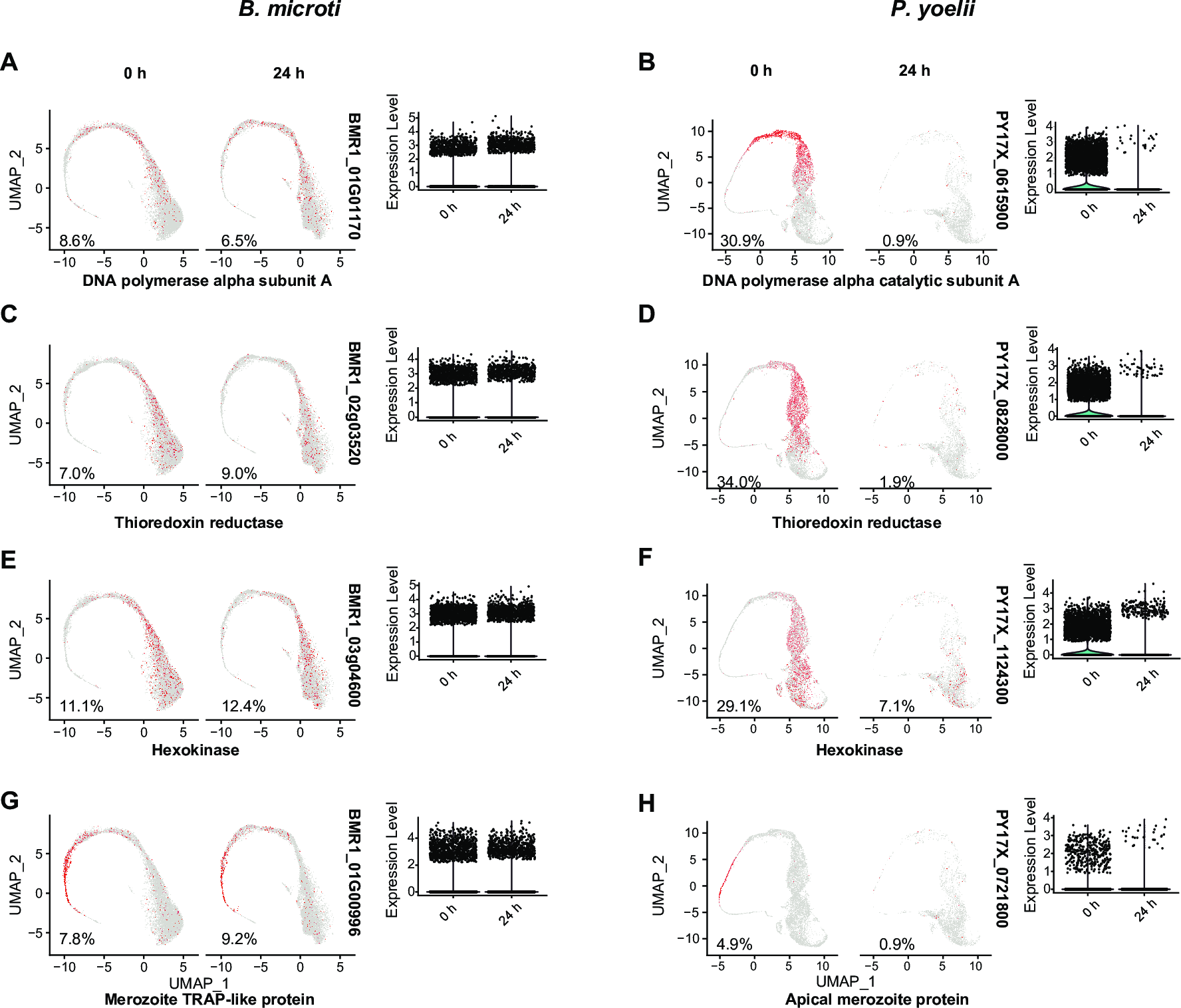

Supplement: Supplementary file 10 — Additional file 10: Fig. S2. UMAP plots showing the sensitivity of Babesia microti and Plasmodium yoelii 17XNL that expressed different genes to 24 h of artemether treatment. a UMAP plots showing the expression characteristic of DNA polymerase alpha subunit Aand the change in the percentage of parasites expressing the gene in the population after 24 h of artemether treatment. b DNA polymerase alpha subunit A. c Thioredoxin reductase. d Thioredoxin reductase. e Hexokinase. f Hexokinase. g Merozoite trap-like protein. h Apical merozoite protein. P < 0.001 for all comparisons. [file 13071_2023_5783_MOESM10_ESM.tif]
